# Establishing a core dossier for multiple regulatory submissions: a case study in the Latin America region

**DOI:** 10.3389/fmed.2023.1102452

**Published:** 2023-05-16

**Authors:** Alva Lucia Alvarez, Irma O. Maisonet, Omar Ruiz, Rebecca S. Lumsden, Ana Paula Evangelista Ferreira, Esther M. Avila Flores

**Affiliations:** ^1^Global Regulatory Affairs International, Pfizer Ltd, Bogotá, Colombia; ^2^Global Regulatory Affairs International, Pfizer Ltd, San Juan, PR, United States; ^3^Global Regulatory Affairs International, Pfizer Ltd, New York, NY, United States; ^4^Global Regulatory Policy and Intelligence, Global Regulatory Affairs, Pfizer Ltd, Tadworth, Surrey, United Kingdom; ^5^Global Regulatory Affairs Operations, Pfizer Ltd, São Paulo, Brazil

**Keywords:** convergence, harmonization, regulatory requirements, acceleration, simplification, divergence, standardization, CTD

## Abstract

The Latin America region comprises several countries that do not follow harmonized regulatory requirements for drug product (DP) marketing authorization applications (MAA), resulting in customized registration dossiers for each country. Here, we established a core dossier for multiple MAA in the Latin America region by examining the similarities between regulatory requirements and reconciling their potential discrepancies through discussions among all national regulatory representatives. The core dossier was used in the submission of a new small molecule, NME1, to nine markets. Assessment of the process included the time to submission; the timing, number, and complexity of questions received; and timing of final national regulatory agencies (NRA) evaluation decisions. The core dossier resulted in an accelerated submission timeline for most markets and earlier receipt of NRA queries from some markets, compared with projections. One round of queries of a low or medium complexity was received from all agencies. The receipt of final NRA evaluation decisions was also accelerated in most markets, compared with the best-case approval timeframes. The core dossier approach was also evaluated against the standard submission of a similar small molecule, NME2. In contrast to the core dossier submission of NME1, a second round of questions, and high-complexity questions were received from two markets for NME2. In conclusion, a core dossier has the potential to simplify the regulatory process for both reviewers and applicants in regions that do not share harmonized regulatory requirements, with a consequential acceleration of DP approvals.

## 1. Introduction

The Latin America and Caribbean (LatAm) region is highly heterogeneous, with diversity in both health systems and patient access to innovative medicinal products (1, 2). National and regional regulatory systems play a key role in addressing these topics. A recent report from the Pan American Health Organization (PAHO) stated, “We will only realize the twin goals of universal access to health and universal health coverage if we ensure access to safe, effective, and quality-assured medicines. Thus, as part of their national health systems, all countries in the Region of the Americas should strive for an effective and efficient regulatory system to regulate and oversee compliance with the highest quality standards for all medical products made available to their populations” (3).

There is a high level of diversity across the LatAm region in both capacity and capability of regulatory systems. A recent study identified that only 23% of PAHO member states (including the United States and Canada) had achieved comprehensive legal and organizational frameworks (4). Over half (57%) of PAHO member states had not fully implemented all the recommended functions for a comprehensive regulatory system and 20% were missing the requisite legal basis and/or organizational structures for regulatory systems (4). This diversity translates into a complex regulatory maze whereby to ensure patient access across the region, the pharmaceutical industry routinely navigates divergent regulatory requirements.

Despite the positive activities driven by the Pan American Network for Drug Regulatory Harmonization (PANDRH), fundamental regulatory activities, such as marketing authorizations and post-approval changes for pharmaceutical products, still follow national legislations and regulatory requirements that have been developed on a country-by-country basis (5). Although this situation is not unique to LatAm, it has led to numerous national regulatory review requirements, which are not always aligned with international guidelines. This offers the opportunity for further convergence to remove any non–science-driven elements that add minimal value for efficient regulatory decision making (6). In the increasingly globalized world of pharmaceutical development, this divergence can lead to delays in filing products, as allocating resources to meet unique national requirements often follows initial global approvals. In the last decade, overall regulatory approval times in some LatAm countries have increased and the variability of review times across the region, influenced by this divergence in requirements, combined with longer, less efficient regulatory reviews can hamper the availability of medicinal products for patients (7).

Based on our long-standing experience in navigating national regulatory systems and the opportunities presented when registering products in LatAm, where a single language is used in over 90% of countries, we aimed to devise an approach whereby a core dossier could be developed for each product, thus reducing the need for multiple translations, and customized to generate as many dossiers as country submissions are required. Here, we describe the development of the core dossier and its impact on streamlining the regulatory process for creating a registration dossier for a MAA in LatAm.

## 2. Methods

In order to file a marketing authorization to a NRA for a DP in multiple countries which do not follow harmonized regulatory requirements, such as the International Council for Harmonization of Technical Requirements for Pharmaceuticals for Human Use (ICH) Common Technical Document (CTD) standards (8), the usual approach is to produce as many customized dossiers as country submissions are needed. Each dossier consists of a hybrid set of components containing: (i) information taken “as is,” for example from the standard CTD sections that were used in the US Food and Drug Administration (FDA) or European Union (EU) initial submissions and (ii) components customized to meet the country-specific requirements that are not covered by the CTD sections. This approach is both labor and resource intense due to the number of customizations needed to create each dossier and the additional work required to update them during the product lifecycle. In this hybrid dossier, the standard sections submitted “as is” are usually those coming from the Clinical (Module 5) and the Nonclinical (Module 4) CTD sections. The customizations occur most commonly in the Quality section (Module 3), due to the high variability observed in this section across the different country regulatory requirements.

An alternative approach could be to utilize a core dossier, which would in turn generate as many dossiers as country submissions were required. This approach could provide a good level of standardization in the customized sections by minimizing the variability in the Module 2 and 3 chemistry, manufacturing, and controls (CMC) components, allowing a single set of components for the assembly of the final dossiers to be submitted.

**Figure 1:**
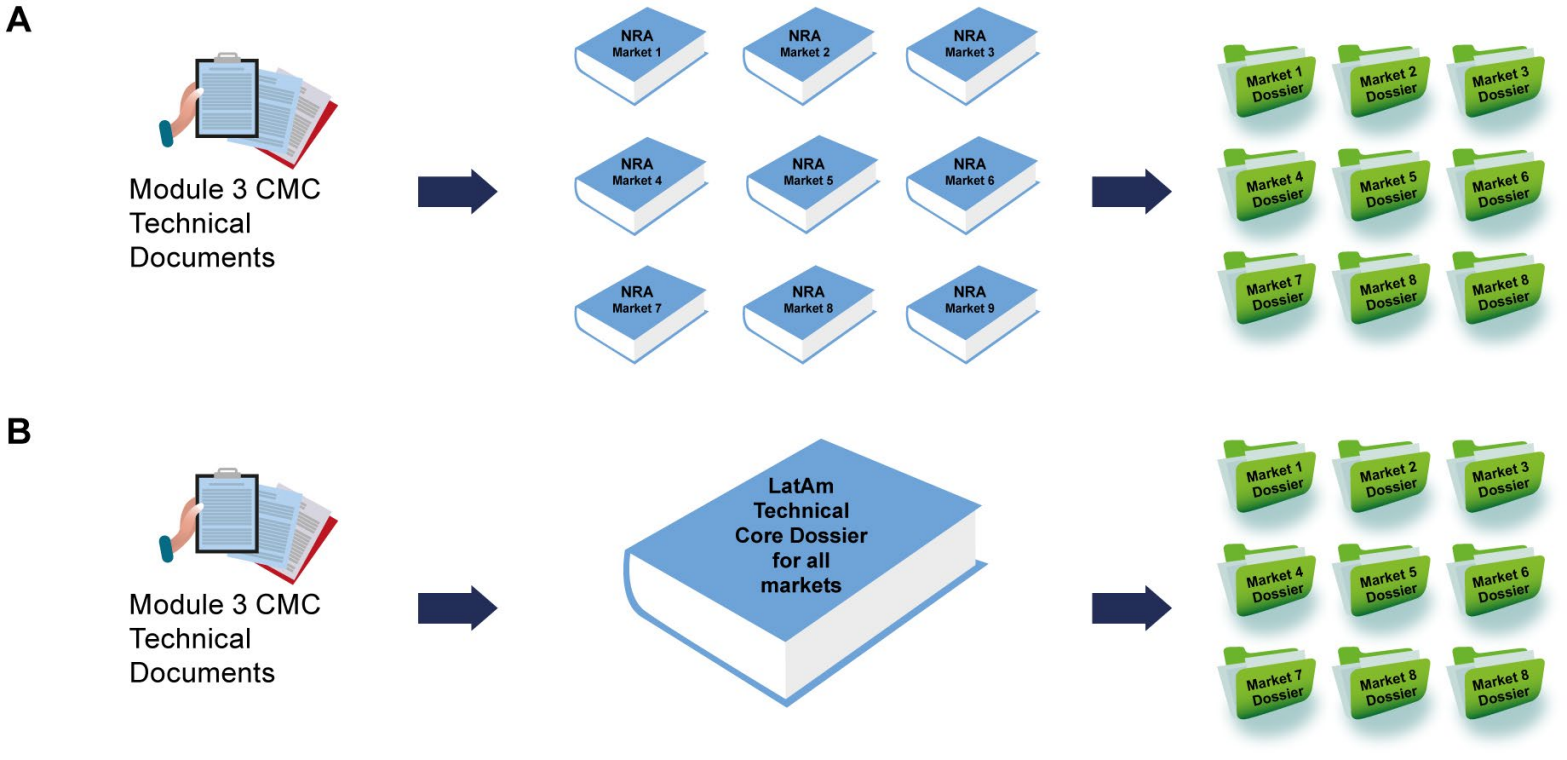
Different approaches to developing the technical components of country-specific dossiers. **(A)** Regular “bespoke” process in which custom documents were produced for each market and **(B)** creation of a LatAm Technical Core Dossier, which was used as the basis of each submission. CMC, chemical, manufacturing, and controls; LatAm, Latin America and Caribbean; NRA, national regulatory authorities.

Regulatory affairs professionals, as experts in regulatory requirements and experienced in technical dialog with regulators, could interpret and analyze all the information in the individual national regulatory requirements. After a careful assessment, they could identify convergences and commonalities across the region, synthesizing various national regulatory requirements into one. In the final stage, all the individual regulatory CTD components together could be transformed into a core document, termed a “LatAm technical core dossier” (Figure 1).

### 2.1. Development of the LatAm core dossier

The similarity in regulatory requirements in LatAm for the registration of new chemical entities (NCEs) and biological products was assessed to identify which countries could potentially follow a core dossier approach. The expected outcome was the generation of a single set of requirements where each component could have a level of detail that allowed its utilization by different countries in the LatAm region.

The individual country regulatory representatives (19 country representatives distributed as follows: 1 Mexico, 2 Central American and Caribbean, 2 Colombia, 8 Peru, Ecuador and Bolivia, 1 Argentina, 1 Uruguay, 1 Paraguay, 1 Chile, 2 Brazil) and authors (5) collected all requirements from the regulations, reviewed and identified commonalities and differences at the national level among the CMC components per product type (NCE and biologics). Following a risk-based approach, the representatives created and agreed a harmonized CMC component set.

### 2.2. Generation of a requirements report

The regulatory requirements for all LatAm markets and the US, selected as the reference global market, were compiled in a centralized database to facilitate the comparison of each requirement across the region. Only CMC components from CTD Modules 2 and 3 were considered. Given the broader number and complexity of the requirements for US, Brazil, Mexico, and Argentina, which already include most of the requirements from the rest of the LatAm countries, they were selected as reference markets for comparison, allowing a higher likelihood of encompassing as many requirements from the other countries as possible. It is important to consider that this was an independent researchers’ initiative and had no participation of the NRAs.

### 2.3. Identification of similarities

**Figure 2:**
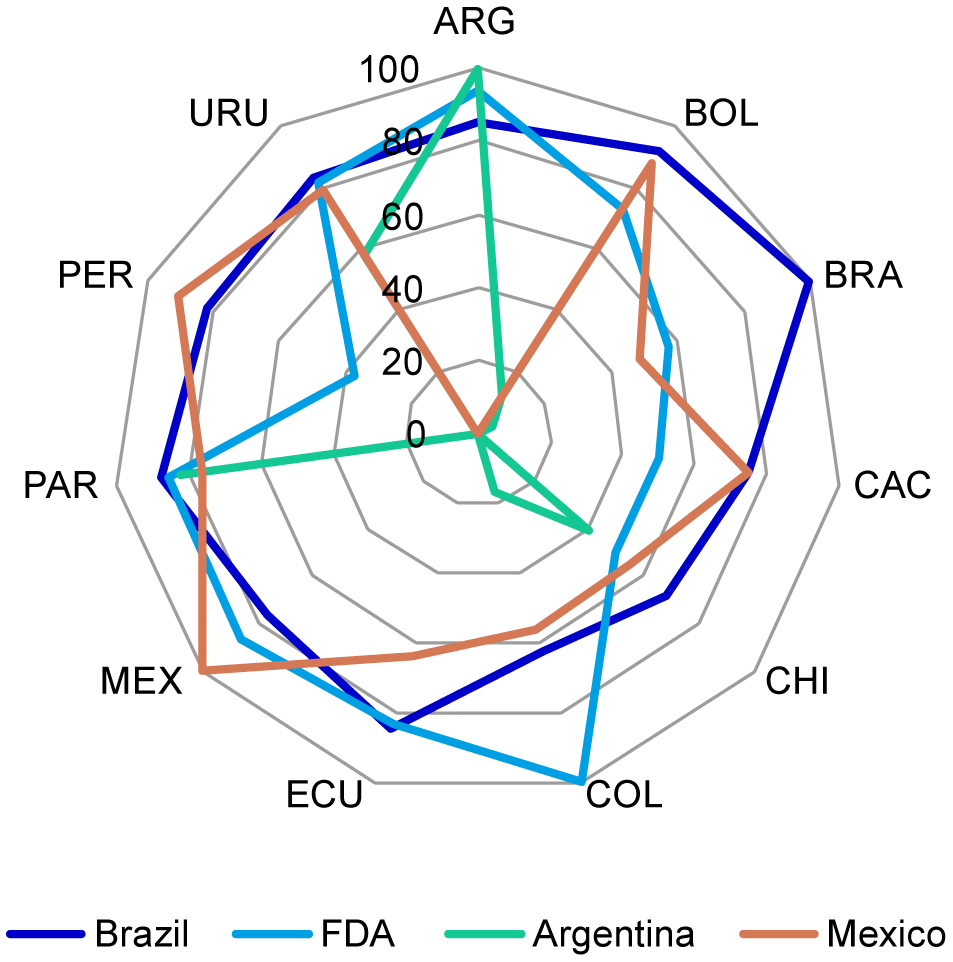
The percentage of CMC components in the reference markets that were considered acceptable for the registration dossier of NCEs in the LatAm markets. The percentage of CMC components in the reference markets that were considered acceptable was plotted for each of the LatAm markets. CMC, chemistry, manufacturing, and controls; FDA, US Food and Drug Administration; LatAm, Latin America and Caribbean; NCE, new chemical entity. Country key: ARG, Argentina; BOL, Bolivia, BRA, Brazil; CAC, Central America and Caribbean; CHI, Chile; COL, Colombia; ECU Ecuador; MEX, Mexico; PAR, Paraguay; PER, Peru; URU, Uruguay.

Chemistry, manufacturing, and controls components from CTD Modules 2 and 3 for NCEs in each market were compared against the regulations from US (FDA), Brazil (Agência Nacional de Vigilância Sanitária – ANVISA), Mexico (Comisión Federal para la Protección contra Riesgos Sanitarios – COFEPRIS), and Argentina (Administración Nacional de Medicamentos, Alimentos y Tecnología Médica – ANMAT), to identify acceptable requirements that could be used “as is” and the components that required discussion or further analysis following identification of major discrepancies. Calculations were performed based on the total number of components required per market (Figure 2). Regulatory representatives identified several discrepancies when comparing the requirements against Argentina and Mexico, although there was greater uniformity across the region with respect to the requirements for US and Brazil (Supplementary Figure S1). Therefore, US and Brazil were selected as the reference market authorities to identify any discrepancies that required further discussion among the team and, if necessary, to discuss with the respective LatAm NRAs to agree on the core dossier approach.

The same exercise that was conducted above for NCEs was performed for biologics. Only the CMC components from the US and Brazil markets were considered as reference, since the evaluation for NCEs determined they were the most appropriate candidates to measure against. The FDA was ultimately selected as the reference agency for biologics, as we identified fewer CMC components requiring further discussion (Supplementary Figure S2).

### 2.4. Requirements analysis: reconciliation of discrepancies

The CMC components originally identified as requiring further discussion or potentially conflicting were analyzed with respect to the following criteria:

- Acceptable or requiring discussion with authorities to obtain acceptance. Market to market differences in the specific CMC requirements that could be acceptable or discussed for acceptance with the authorities.
- “Nice-to-haves” or “must-haves.” Requirements at potential risk of rejection, or likely to receive queries from the authority were categorized as nice to haves or must haves.
- “As is.” Requirements that could be taken “as is” in the reference market or supplemented with complementary information to be more acceptable for the authorities. This level of customization must be accepted for all markets to achieve the convergence approach.

**Figure 3:**
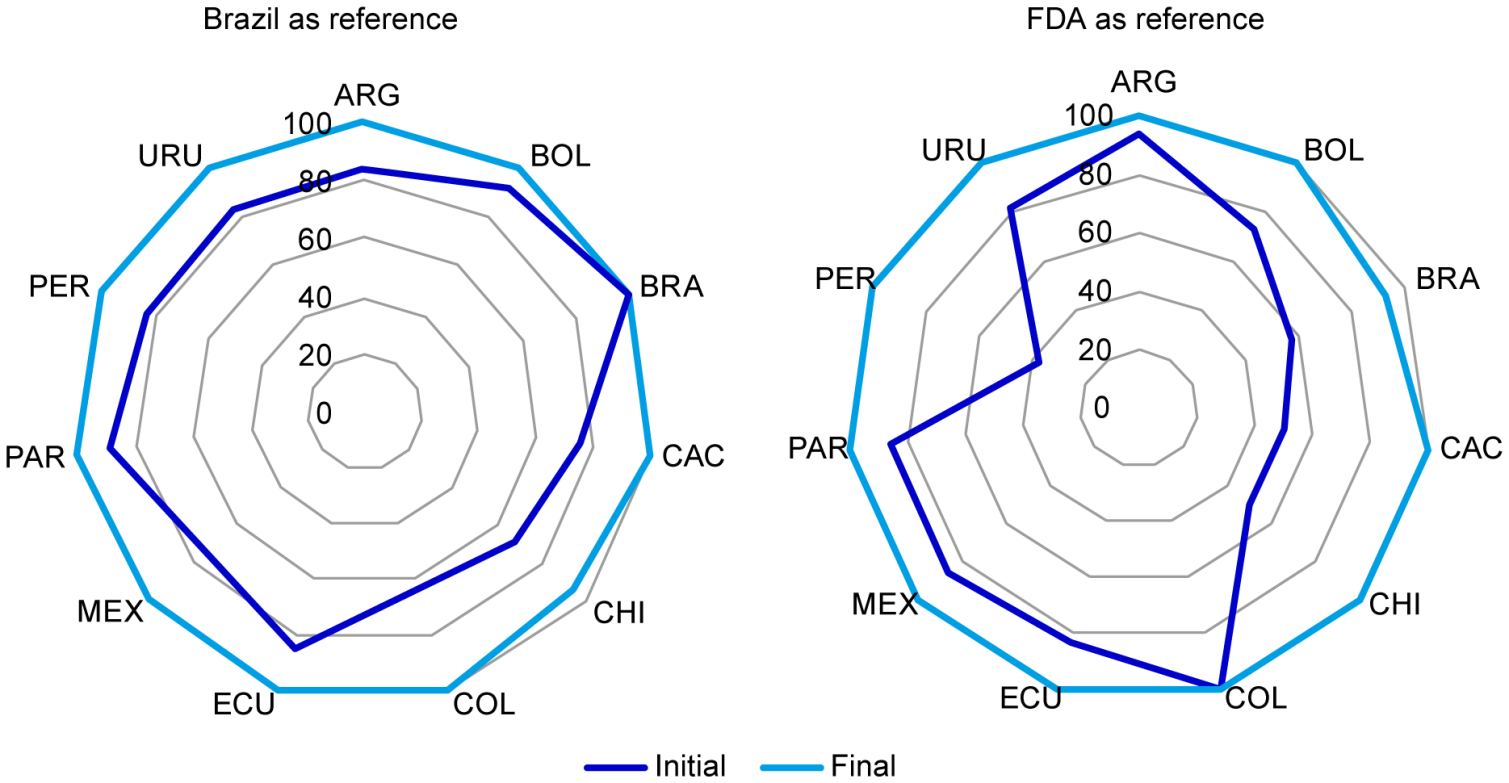
Chemistry, manufacturing, and controls (CMC) components in Brazil and US dossiers for NCEs that were considered acceptable before and after the harmonization exercise. The initial and final status of **(A)** Brazil and **(B)** US reference markets for NCEs was plotted. In each market, the percentage of acceptable CMC components increased after reconciliation of discrepancies in format and/or content through discussion among representatives from all markets as stated in section 2.1. CMC, chemistry, manufacturing, and controls; FDA, Food and Drug Administration; NCE, new chemical entity. Country key: ARG, Argentina; BOL, Bolivia, BRA, Brazil; CAC, Central America and Caribbean; CHI, Chile; COL, Colombia; ECU Ecuador; MEX, Mexico; PAR, Paraguay; PER, Peru; URU, Uruguay.

**Figure 4:**
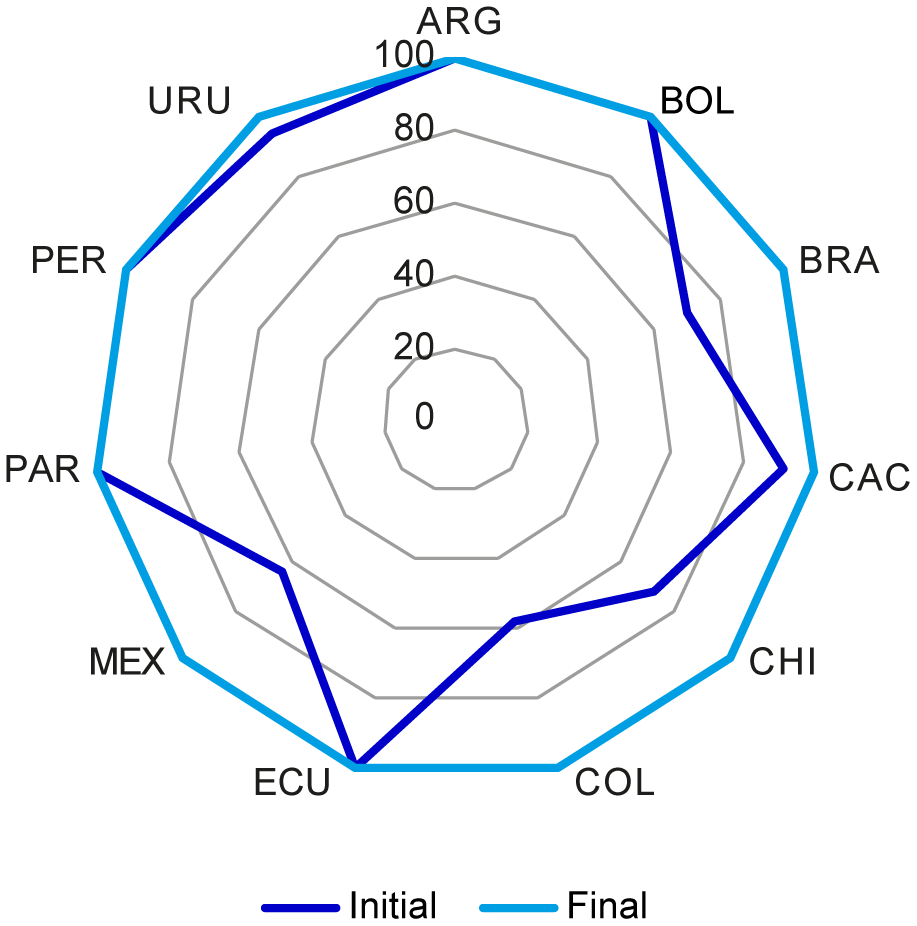
Chemistry, manufacturing, and controls (CMC) components in the US dossier for biologics that were considered acceptable before and after the reconciliation of discrepancies. The initial and final status of the US reference market for biologics was plotted. In each market, the percentage of acceptable CMC components increased after reconciliation of discrepancies through discussion among representatives from all markets as stated in section 2.1. CMC, chemistry, manufacturing, and controls; FDA, Food and Drug Administration. Country key: ARG, Argentina; BOL, Bolivia, BRA, Brazil; CAC, Central America and Caribbean; CHI, Chile; COL, Colombia; ECU Ecuador; MEX, Mexico; PAR, Paraguay; PER, Peru; URU, Uruguay.

For each reference market, the percentage of acceptable CMC components for NCEs (Figure 3) and biologics (Figure 4) increased after discussions among the representatives from all markets and the reconciliation of discrepancies according to the above criteria.

### 2.5. Deliverables and data validation

Following the reconciliation of discrepancies, the harmonized single set of requirements were compiled under the following headings:

- Product type.
- Submission category.
- List of core global CMC (GCMC) components.
- Harmonized and detailed description of the requirement.
- Individual market needs of the requirement.

Given the amount of data included in the deliverable and its importance for the next steps of the project, the resulting table was validated again by each regulatory representative to ensure that all agreed statements for each of the requirements were complete and accurate.

## 3. Results

### 3.1. Application of a core dossier for multiple regulatory submissions

A submission with the pilot dossier of a marketing authorization was planned and executed to investigate how the core dossier performed in the real business environment. Following discussions regarding the creation of the core dossier, NRAs were not informed of the source of information included in the submission with the pilot dossier. The company submission plan for synthetic new small molecules was evaluated to identify an asset that best suited this intention. To maintain proprietary information, confidentiality, and for the purpose of this article, the molecule selected was referred to as new molecular entity (NME)1. The selection of NME1 was based on:

- Number of potential markets impacted. The pilot should ideally include all nine markets that have been identified in the upcoming submission plan for NME1 to the NRAs (Colombia, Costa Rica, Dominican Republic, Ecuador, Guatemala, Panama, Paraguay, Peru, Uruguay), spanning large, medium, and small populations, to test if the core dossier could be used as a “one fits all” solution. The rest of the LatAm countries (Argentina, Mexico, Chile and Brazil) were not included in the pilot dossier as they were already submitted to the NRAs.
- Submission dates spanned across at least 6 months. Benefit from the pilot should be tangible against company goals to reduce healthcare disparities and have an equitable approach for the region. Acceleration must be evident by choosing projects where small markets show a clear lag in submission timelines, compared with medium and large markets.
- First submission date at least 6 months in the future. After selection of the asset, additional processes were needed, such as management endorsement and incorporation into the global regulatory product strategy and training. Thus, choosing projects with submission dates earlier than 6 months in the future may have led to rushed implementation and unsuccessful results.
- No GCMC activities started for any market. Though this criterion was not essential, it was preferred to select products where no GCMC components authoring had been initiated. This would avoid repetition of work completed by GCMC teams in Module 2 and 3 components previously undertaken for an individual market.

**Figure 5:**
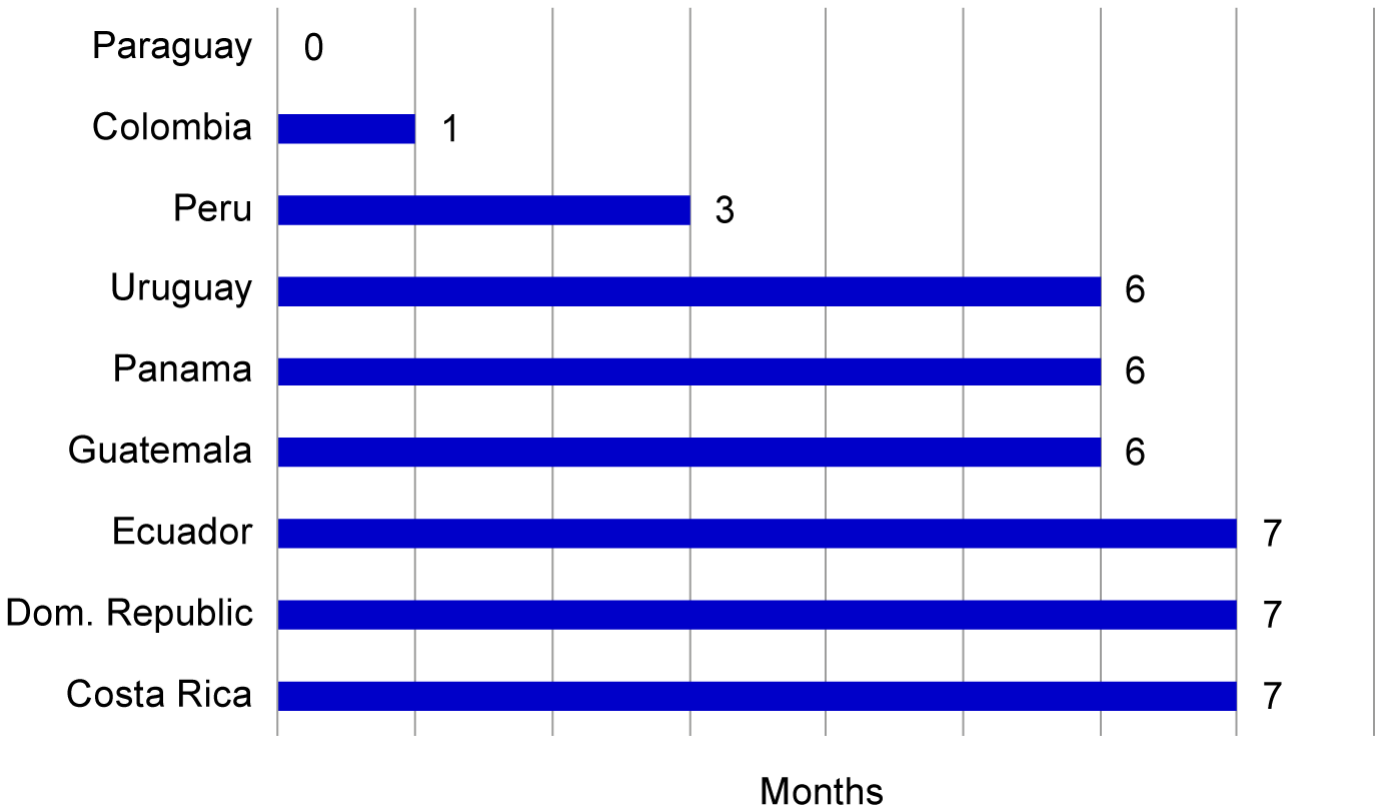
The effect of a core dossier on submission plan acceleration. The submission acceleration (months) for each market was plotted relative to the initial projected data. These data indicated that the core dossier approach accelerated submission in the majority of markets.

The use of the core dossier in consolidating the CMC related module requirements for the region optimized the time for planning meetings. A kick-off meeting was established with the designated countries, simplifying multiple meetings for each country into a single combined session. The filing plan was aligned for seven markets, and 2 markets were scheduled to be filed 4 months later. The activities of authoring multiple CMC components into one virtual document facilitated a more expeditious dispatch to the markets, potentially advancing the submission schedule. When the projected submission plan without a core dossier was compared with the plan using this approach, we observed an acceleration of up to 7 months in the submission period for participating countries (Figure 5).

Upon submission to NRAs, the application built applying core dossier was evaluated from the perspective of the observations received by the different regulatory agencies during their review process. The aspects considered were the following:

- Timing: When the NRA query was expected versus the actual date of query issuance.
- Complexity: How much effort from above country support was required to furnish a response to the regulatory agency (see Supplementary Figure S3).

The NRA query reception date was tabulated (Table 1), and graphs were created depicting the number of letters, as well as the number and complexity of questions received. The letters received from the authorities were issued, one per dose strength/license/registration for each country. Because NME1 had three dose strengths, three letters per country were handled. There were no questions received for Module 3 from the regulatory agencies of Panama or the Dominican Republic (Supplementary Figure S4). The number of questions received varied for each NRA. Questions were classified into three groups: the same set of questions for each license (Ecuador and Peru), specific questions per license (Uruguay), and both shared and specific questions per license (Colombia, Costa Rica, and Guatemala) (Supplementary Figure S5). It should be noted that this behavior may have been influenced by the number of auditors reviewing the file and/or by the content information in the file.

**Table 1:** National regulatory agencies (NRA) query timelines for core dossier first submissions.

Country	Projected query time (weeks)	Actual query time (weeks)
Colombia	57	48
Costa Rica	12	17
Dominican Republic	4	4^a^
Ecuador	12	31^a^
Guatemala	4	8
Panama	16	7^a^
Peru	24	27

^a^First query was not CMC-related. The projected query receipt time was compared with the actual receipt time of the first query in the country offices. For some countries, the actual date of receipt was ahead of the projected date. Paraguay and Uruguay not included since they were in the second wave of submissions. CMC, chemistry, manufacturing, and controls; NRA, national regulatory authority.

**Figure 6:**
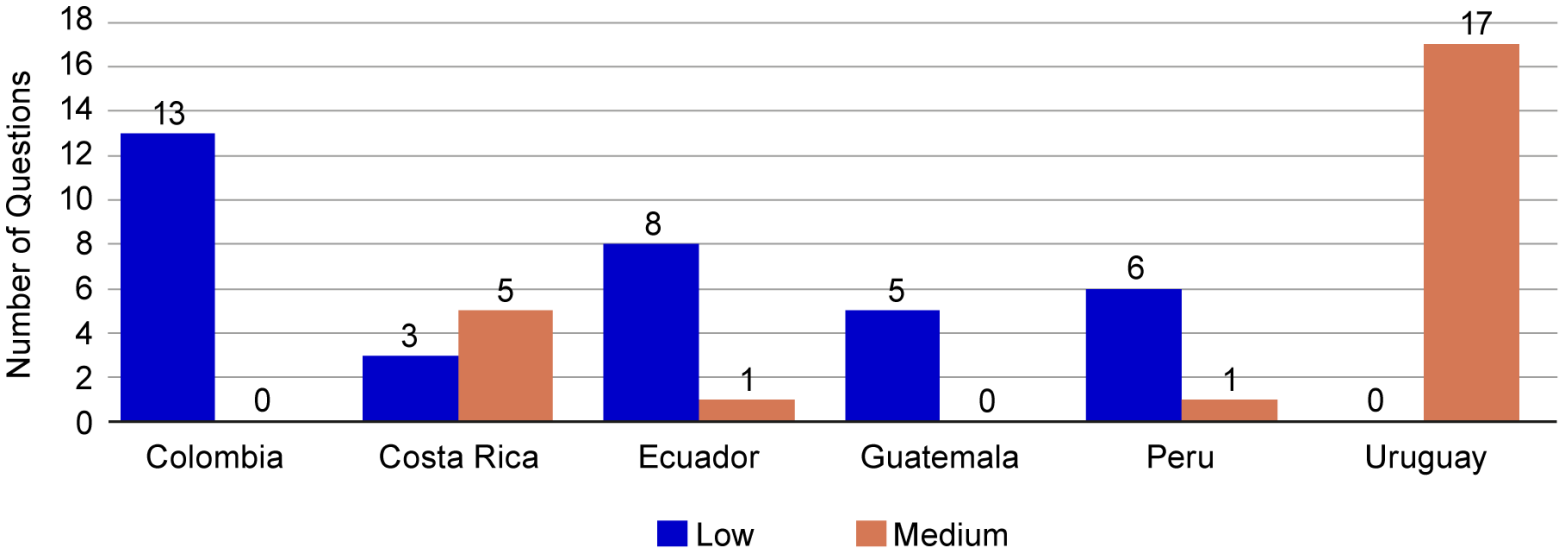
Complexity of questions received for NME1. The CMC questions for NME1 submission were classified as low, medium, or high complexity according to the additional creation of documentation required to provide an answer. All the questions received from the core dossier submission of NME1 were of low (blue bars) or medium (orange bars) complexity. CMC, chemical, manufacturing, and controls; NME, new molecular entity.

One important benefit of the core dossier could be the simplification of the process for regulatory submissions. The harmonization of components could assist regulatory agencies to ensure a smooth review process and improve comprehension of the CMC content. To assess this point, we considered the complexity of questions made by the auditors in the evaluations. These questions were classified as low, medium, or high complexity, depending on whether additional documentation was required to provide an answer (Supplementary Figure S3). The CMC questions evaluated for NME1 in most of the LatAm markets included in the submission utilizing the core dossier were cataloged as medium and low complexity; no high-complexity questions were received (Figure 6). For Uruguay, the questions were of medium complexity, and in both Colombia and Guatemala the CMC questions were of low complexity. For Costa Rica, Ecuador, and Peru, both low- and medium-complexity questions were received. It should be noted that no questions led to the revision of the set of components established in the CMC core dossier. Only in Module 3 Regional section 3.2R, a local form was revised to add a minor detail to clarify the information reported.

**Figure 7:**
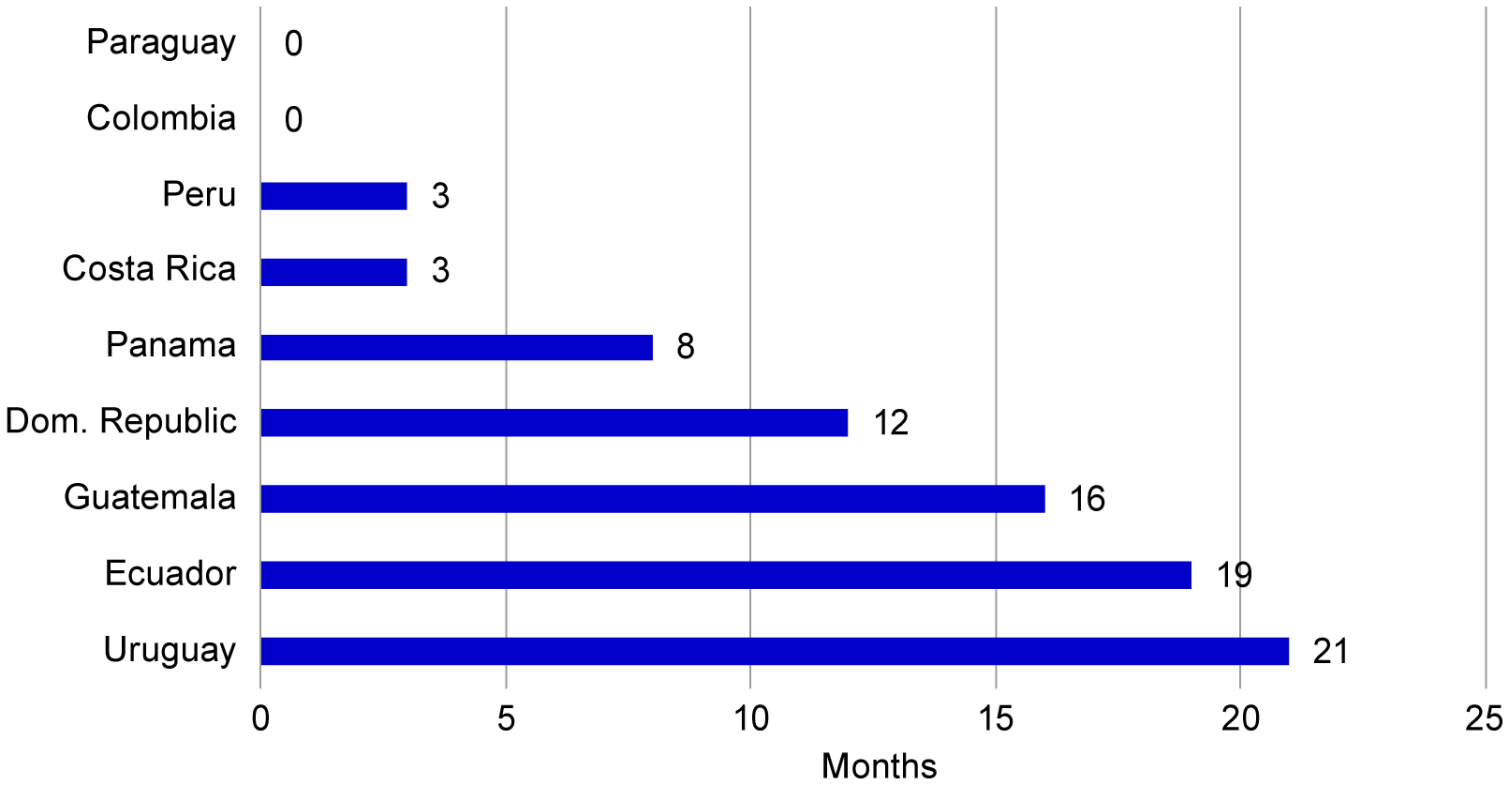
Approval acceleration of NME1 compared with best-case estimates. Final NRA evaluation decisions for NME1 were received between 0 and 21 months ahead of the projected date, suggesting that use of a core dossier resulted in accelerated approval timeframes. NME, new molecular entity; NRA, national regulatory agency.

The use of a core dossier not only resulted in an accelerated submission schedule for NME1, but also positively impacted the NRA evaluation period. The NRA final decisions were received up to 21 months ahead of the estimated best-case approval timeframes for each market (Figure 7). This acceleration may be associated with bringing forward the original submission dates as established in the original operating plan, simplification of data content, reduction in the amount of country specific CMC documents to be created and less complex NRAs questions when using a core dossier.

### 3.2. Comparison of regulatory submissions with and without a core dossier

To evaluate the potential benefits of a core dossier, the regulatory submission of NME1 to NRAs was compared to that of a second asset, NME2, that did not use a core dossier. The submission of NME2 was for the same product type, dosage form, during a similar submission period to the same countries as NME1. As for NME1, NRAs were not informed of the source of information included in the NME2 submission. NME2 had two dose presentations, whereas NME1 had three. Similar to the observations for NME1, the letters received from the authorities for NME2 were a letter/dose strength/registration. However, in two countries (Guatemala and Uruguay) NME2 received a second round of questions by the authority (Supplementary Figure S6).

**Figure 8:**
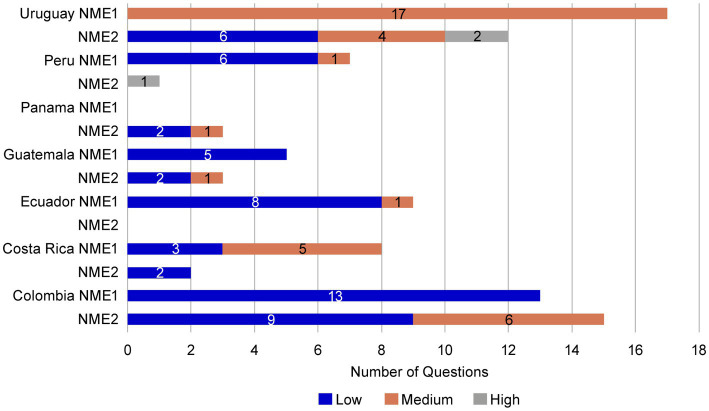
Complexity of questions received for NME1 and NME2. Like those received for NME1, most questions received for NME2 were of low (blue bars) or medium (orange bars) complexity. However high complexity questions (gray bars) were received from Peru and Uruguay for NME2. No high-complexity questions were received for NME1. NME, new molecular entity.

Most of the questions received from the NRA agencies for NME2 were shared for all dose strengths, except in Colombia, where there were also specific questions by dose strength. Ecuador and Dominican Republic NRAs did not issue CMC questions for NME2 (Supplementary Figures S6, S7). The questions received during the NME2 NRAs reviews were generally identified as low to medium complexity. However, high-complexity questions were received from NRA reviews in Peru and Uruguay (Figure 8).

## 4. Discussion

Convergence and harmonization continue to play a fundamental role in strengthening regulatory systems across the LatAm region. We support ongoing efforts to drive convergence at both the regional and global level, particularly with respect to international guidelines. Whether to facilitate regulatory reliance or address health emergencies, convergence with science-based regulations can ultimately facilitate regulatory submissions and expedite patient access to innovative medicines. The LatAm core dossier was a practical demonstration that convergence in LatAm is achievable; if not for a full dossier, at least for important sections within it. We have achieved practical convergence by producing a core set of data that was able to meet the requirements in multiple markets for the molecule NME1, submitted in nine countries with different regulatory requirements. At the time of writing, NME1 has been granted approval in seven countries and is undergoing regulatory review in two countries.

The NRAs regulatory reviews for NME1 demonstrated several potential benefits of a core dossier, including accelerated submission plans (in comparison to the initial projections), earlier receipt of queries from regulatory reviews (compared with projected dates), a decreased complexity of questions received (compared with reviews of NME2, which used a standard submission), and accelerated approvals (compared with projected best-case approval timeframes for each market).

Comparison of regulatory reviews for NME1 and NME2 revealed that the core dossier resulted in a greater proportion of low- to medium-complexity questions received, as no high-complexity questions were received for core dossier submissions. To evaluate which factors could have contributed to the low- to medium-complexity level of the questions received, those that may have affected the regulatory review should be considered. Presentation of data taken from a core dossier according to a market’s particular interests and concerns, and a focus on harmonizing data according to the market’s set of standards may contribute to their smooth evaluation. The data from this study suggest there is no evidence that the core dossier increases the complexity of questions received during regulatory review, and it is likely that the simplification and harmonization of the CTD Module 2 and 3 CMC components assists the review of the application by generating clearer content.

This study demonstrates the potential benefits and reproducibility of a core dossier for multiple submissions in the LatAm region for the applicants. However, the limitations of the study should also be considered when interpreting the findings. The first potential limitation is the comparison of the core dossier submission to only one standard submission. Further regulatory submissions using a core dossier in LatAm are required to investigate the potential benefits of this approach. However, it should be noted that NME1 and NME2 represent similar molecules, submitted to the same markets in a similar timeframe, providing a reasonable initial comparison of the core dossier to a standard submission. It is also important to note that applicant discussions regarding the development of the core dossier did not influence the NRAs regulatory review of NME1 or NME2, as the NRAs were not informed of the source of information included in either submission. Secondly, the influence of the pandemic period caused by COVID-19 on the evaluation of these applications should be acknowledged, as it might have been a factor that impacted the assessment process by the reviewers Third, elements at the agency level specific to each country, such as workload, office size, personnel level of experience and prioritization should also be considered when analyzing the results of this study. Finally, the criteria applied during the selection of NME1 and NME2 as candidate molecules for this study may have influenced the results produced. This point could be further evaluated using other medicines that typically have shorter submission times across the region.

In conclusion, a core dossier was generated and utilized to produce nine registration dossiers that were able to fulfill the NRAs regulatory requirements in countries that have no harmonized regulations. This study demonstrates that regulatory convergence in the pharmaceutical industry is possible and has the potential to simplify the process for both reviewers and applicants, with the consequential acceleration of approvals and availability of new medicines to patients.

## Data availability statement

The datasets presented in this article are not readily available because upon request, and subject to review, Pfizer will provide the data that support the findings of this study. Requests to access the datasets should be directed to https://www.pfizer.com/science/clinical-trials/trial-data-and-results.

## Author contributions

AA, IM, OR, and EA were involved in the conception and design of the study, and in the performance of the research. All authors were involved in analysis, interpretation of the data, and drafting of and revising the manuscript, approved the final version for submission, and read and agreed to the published version of the manuscript.

## Funding

This study was sponsored by Pfizer. The funder was involved in the study design, collection, analysis, interpretation of data, preparation of the manuscript, and decision to publish.

## Conflict of interest

AA, IM, OR, AF, and EA are full-time employees, and hold stock or stock options in Pfizer. RL was employed by Pfizer during the development of the manuscript and holds stock or stock options in Pfizer.

The authors declare that this study received funding from Pfizer. The funder was involved in the study design, collection, analysis, interpretation of data, preparation of the manuscript, and decision to publish.

## Publisher’s note

All claims expressed in this article are solely those of the authors and do not necessarily represent those of their affiliated organizations, or those of the publisher, the editors and the reviewers. Any product that may be evaluated in this article, or claim that may be made by its manufacturer, is not guaranteed or endorsed by the publisher.

## Supplementary material

The Supplementary material for this article can be found online at:

https://www.frontiersin.org/articles/10.3389/fmed.2023.1102452/full#supplementary-material

## Abbreviations

CMC: chemistry, manufacturing, and controls
CTD: common technical document
FDA: US Food and Drug Administration
GCMC: global chemistry, manufacturing, and controls
ICH: International Council for Harmonization of Technical Requirements for Pharmaceuticals for Human Use
LatAm: Latin America and Caribbean
MAA: marketing authorization application
NCE: new chemical entity
NME: new molecular entity
NRA: national regulatory agency
PAHO: Pan American Health Organization
PANDRH: Pan American Network for Drug Regulatory Harmonization

## References

[1] Mejia A Gilardino R Kristensen FB Garrison LP Roundtable IHTALA. Value-based pricing in Latin America: how far away are we? Value health reg. Issues. (2018) 17:219–23. doi: 10.1016/j.vhri.2018.09.007, PMID: 30528780

[2] Ruano AL Rodriguez D Rossi PG Maceira D. Understanding inequities in health and health systems in Latin America and the Caribbean: a thematic series. Int J Equity Health. (2021) 20:94. doi: 10.1186/s12939-021-01426-1, PMID: 33823879 PMC8023548

[3] Pan American Health Organization. Regulatory system strengthening in the Americas. Lessons learned from the National Regulatory Authorities of regional reference. Washington DC: PAHO, World Health Organization (2021).

[4] Preston C Freitas Dias M Pena J Pombo ML Porras A. Addressing the challenges of regulatory systems strengthening in small states. BMJ Glob Health. (2020) 5:e001912. doi: 10.1136/bmjgh-2019-001912, PMID: 32180997 PMC7053784

[5] Pombo ML Porras A Saidon PC Cascio SM. Regulatory convergence and harmonization: barriers to effective use and adoption of common standards. Rev Panam Salud Publica. (2016) 39:217–25. PMID: 27706409

[6] O'Brien J Lumsden RS Diehl DH Macdonald JC. Building a better approach for the benefit of patients: 10 pillars to strengthen regulatory review systems globally. Ther Innov Regul Sci. (2020) 54:283–92. doi: 10.1007/s43441-019-00055-9, PMID: 32072580

[7] Patel P. McAuslane N. Liberti L. (2019). CIRS RD briefing 71: trends in the regulatory landscape for the approval of new medicines in Latin America. London, UK: Centre for Innovation in regulatory science (CIRS). Available at: https://cirsci.org/wp-content/uploads/2020/02/CIRS-RD-Briefing-71-Trends-in-the-regulatory-landscape-Latin-America.pdf

[8] European Medicines Agency. International Council for Harmonisation of Technical Requirements for Pharmaceuticals for Human Use, ICH harmonised guideline organisation of the common technical document for the registration of pharmaceuticals for human use m4(r4) (2021). Available at: https://www.ema.europa.eu/en/documents/scientific-guideline/ich-guideline-m4-r4-common-technical-document-ctd-registration-pharmaceuticals-human-use_en.pdf

