# Supplementary material for: Establishing a core dossier for multiple regulatory submissions: a case study in the Latin America region
Source: Front Med (Lausanne). 2023 May 16;10:1102452. doi: 10.3389/fmed.2023.1102452 (PMC10229127; doi:10.3389/fmed.2023.1102452)
Supplement: Supplementary file 1 [file Data_Sheet_1.pdf]

Supplementary Figure S1. Percentage of CMC components from the reference markets that were considered acceptable, requiring further analysis or potentially conflicting for the registration dossier of NCEs in the LatAm markets

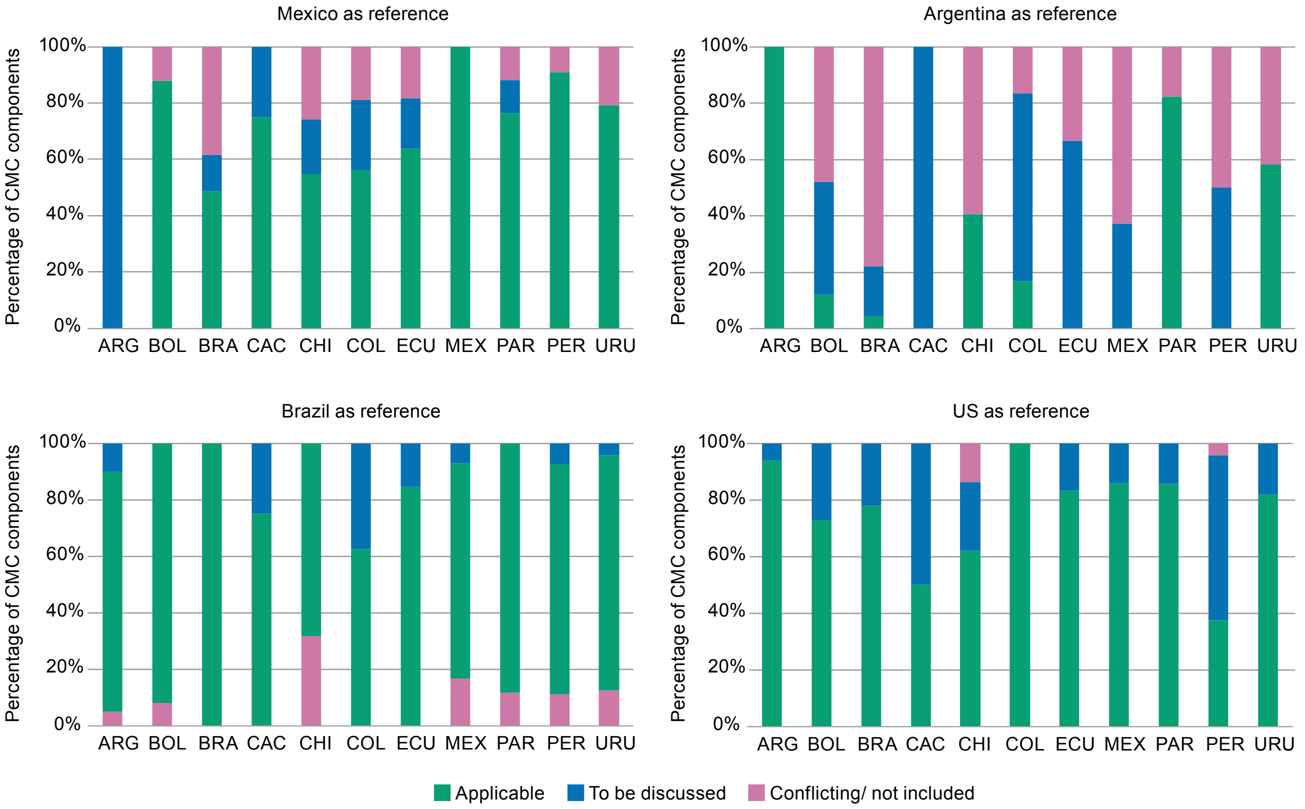

The percentage of components per country that were acceptable (green bars), those to be discussed further (blue bars) and those with major conflicts (pink bars) were plotted for NCEs. The US FDA and Brazil reference markets had the most acceptable requirements across the region.

CMC, chemical, manufacturing, and controls; FDA, Food and Drug Administration; LatAm, Latin America and Caribbean; NCE, new chemical entities.

Country key:

ARG, Argentina; BOL, Bolivia, BRA, Brazil; CAC, Central America and Caribbean; CHI, Chile; COL, Colombia; ECU Ecuador; MEX, Mexico; PAR, Paraguay; PER, Peru; URU, Uruguay

Supplementary Figure S2. Percentage of CMC components from the reference markets that were considered acceptable, requiring further analysis or potentially conflicting for the registration dossier of biologics in the LatAm markets

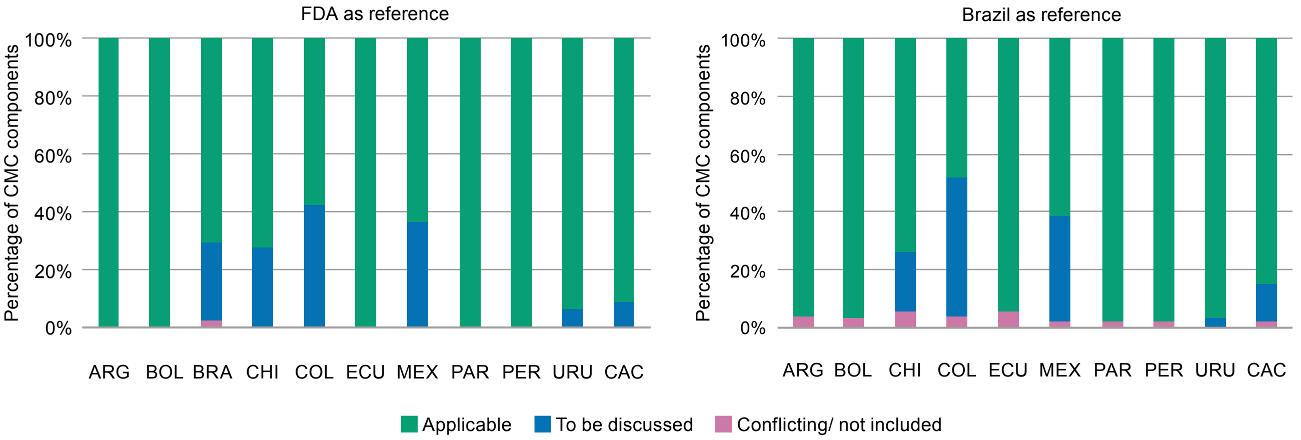

The percentage of components per country that were acceptable (green bars), those to be discussed further (blue bars) and those with major conflicts (pink bars) were plotted for NCEs. The US and Brazil reference markets had the most acceptable requirements across the region.

CMC, chemical, manufacturing, and controls; FDA, Food and Drug Administration; LatAm, Latin America and Caribbean; NCE, new chemical entities.

Country key:

ARG, Argentina; BOL, Bolivia, BRA, Brazil; CAC, Central America and Caribbean; CHI, Chile; COL, Colombia; ECU Ecuador; MEX, Mexico; PAR, Paraguay; PER, Peru; URU, Uruguay

Supplementary Figure S3. Complexity criteria for NRA query classification. The questions received from NRA reviews were classified as high, medium, or low complexity according to the effort from above country support that was required to provide a response.

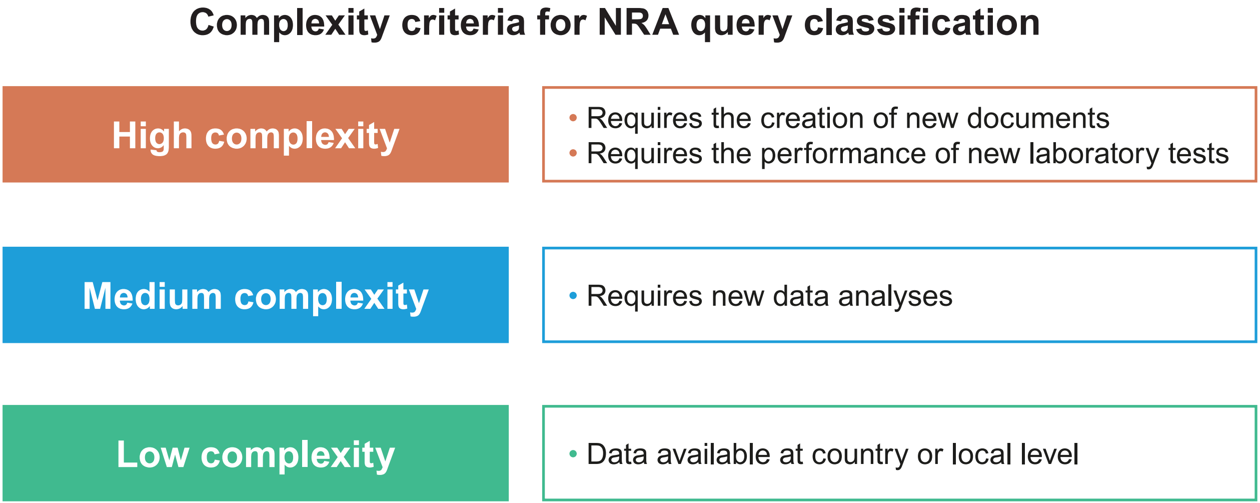

Lab, laboratory; NRA, national regulatory authorities.

Supplementary Figure S4. Number of letters received from each country for NME1 submission

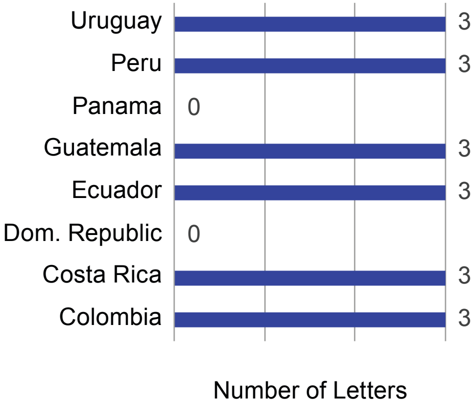

For the majority of countries, one letter per concentration/license/registration of NME1 was received. No questions for Module 3 were received from Panama or the Dominican (Dom.) Republic.

CMC, chemical, manufacturing, and controls; LatAm, Latin America and Caribbean; NME, new molecular entity.

Supplementary Figure S5. Classification of questions per license for NME1 submission

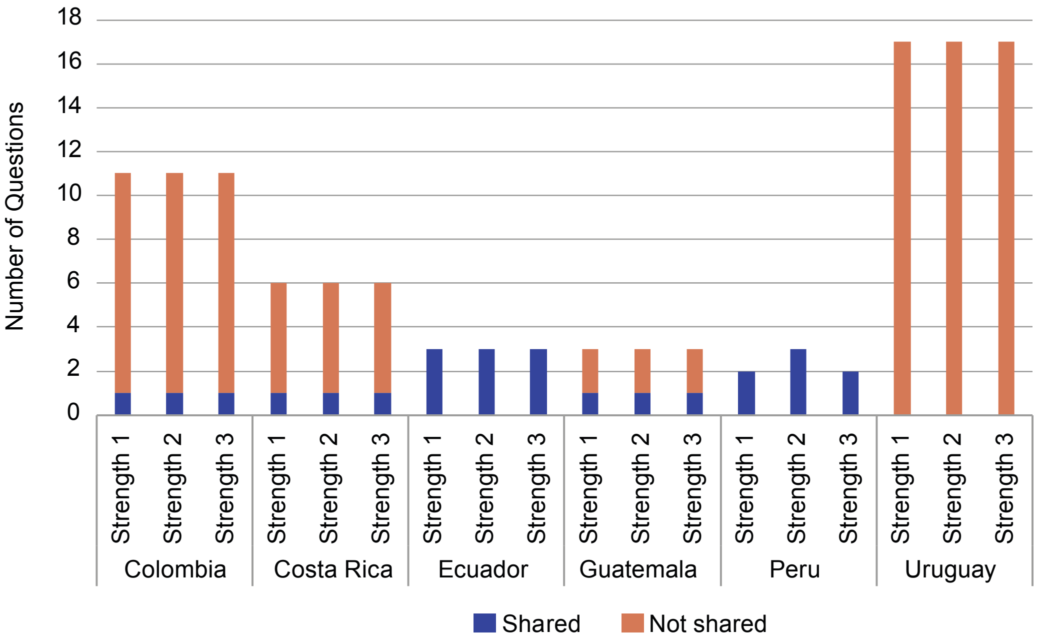

Questions from regulatory agencies were classified into those shared between licenses (orange) and specific questions per license (blue) for each dose strength. This created three groups of markets, those with the same set of questions for each license (Uruguay); specific questions per license (Ecuador and Peru) and both shared and specific questions per license (Colombia, Costa Rica, Guatemala).

NME, new molecular entity.

Supplementary Figure S6. Number of letters received from each country for NME1 and NME2. (A) For NME1, which had three dose presentations, a letter was received per concentration/registration from most countries. (B) For NME2, which had two dose presentations, a letter per concentration/registration was received from most countries. However, a second round of questions was received from Guatemala and Uruguay.

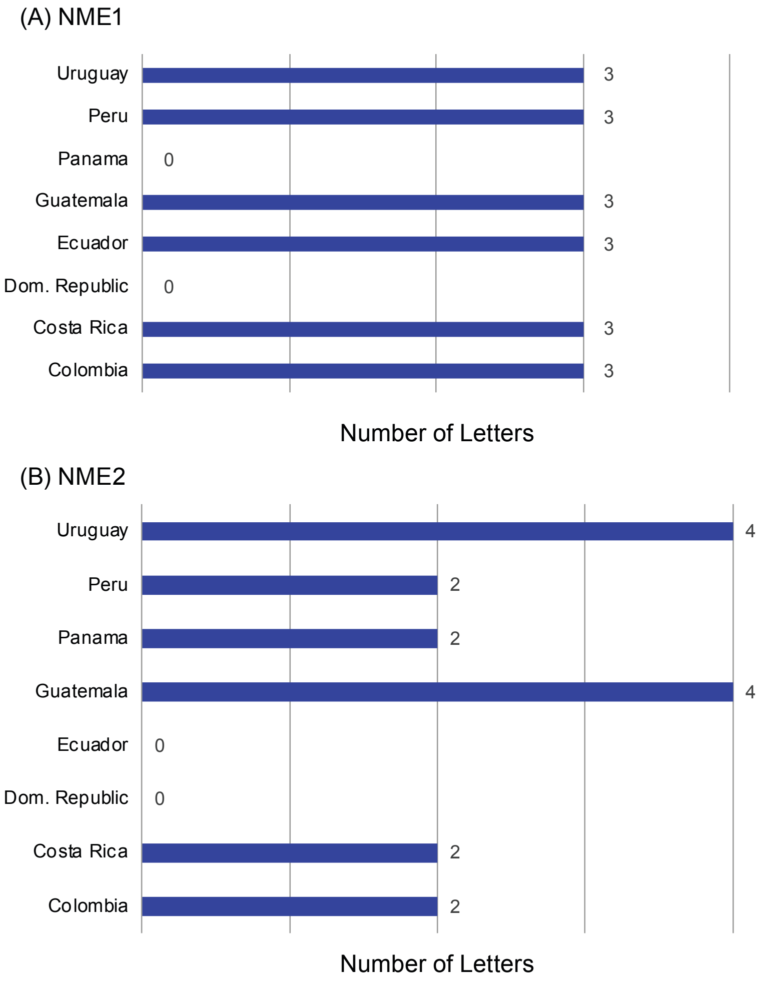

Dom., Dominican (Dom.) Republic; NME, new molecular entity.

Supplementary Figure S7. Classification of questions per license for NME1 and NME2

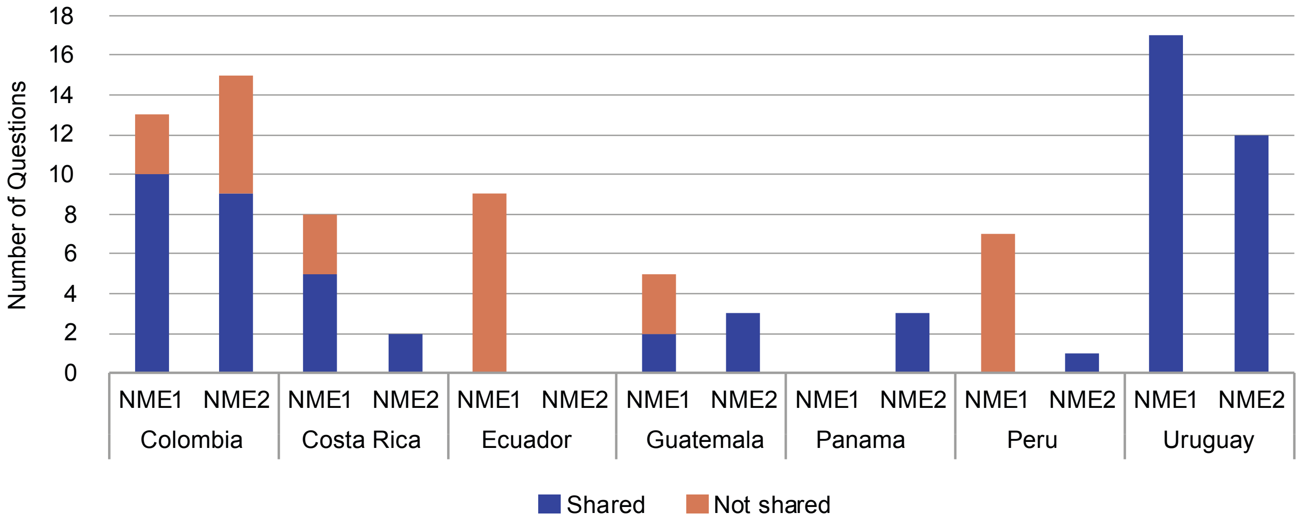

Most questions received for NME2 were shared for all concentrations (blue bars), except those received from Colombia, where there were also specific questions per concentration (orange bars).

NME, new molecular entity.
